# Social Risk Factors of Transportation PPP Projects in China: A Sustainable Development Perspective

**DOI:** 10.3390/ijerph15071323

**Published:** 2018-06-24

**Authors:** Jingfeng Yuan, Wei Li, Jiyue Guo, Xianbo Zhao, Mirosław J. Skibniewski

**Affiliations:** 1 Department of Construction and Real Estate, School of Civil Engineering, Southeast University, Nanjing 210096, China; 230179099@seu.edu.cn (W.L.); 220161160@seu.edu.cn (J.G.); 2 School of Engineering and Technology, Central Queensland University, 400 Kent Street, Sydney, NSW 2000, Australia; b.zhao@cqu.edu.au; 3 Department of Civil and Environmental Engineering, University of Maryland, College Park, MD 20742, USA; mirek@umd.edu

**Keywords:** public private partnerships (PPPs), social risk factors (SRFs), sustainable development, confirmatory factor analysis (CFA), questionnaire survey

## Abstract

Public-private partnerships (PPPs) have become increasingly important in improving the sustainability of society in China, with transportation being the largest investment area. However, the Social Risk Factors (SRFs) of transportation PPPs in China, which serve as a useful tool for distinguishing strengths and weaknesses for effective social risk management (SRM), have not been clearly identified. A conceptual model including 3 risk dimensions and 15 SRFs was proposed to mitigate social risks and improve the social sustainability of transportation PPP projects. A questionnaire survey conducted to investigate stakeholders’ opinions on the proposed SRFs demonstrated that all the SRFs were important. The SRFs can be used to evaluate social risks from economic, environmental, and social dimensions. Confirmatory factor analysis (CFA) verified the classification of the SRFs and indicated that all the risk dimensions contributed to social risks. The social and environmental impacts on social sustainability may contribute more to the generation of social risks. Furthermore, the concept of people-first PPPs was proposed to reduce social risks from the perspective of different stakeholders, with the interactions among different stakeholders being prioritized. The identified SRFs and their relationships can improve our understanding of SRM in the delivery of social sustainability and improve social resilience.

## 1. Introduction

Since 2014, the Chinese government has widely adopted Public Private Partnerships (PPPs) to promote urbanization, provide high-quality services and public goods, and help to reduce fiscal pressure [1]. According to Bridata [2], 3774 PPP projects were procured, and investments reached 5570 billion CNY in the period between January 2014 and June 2017. Among these projects, at least 20% are in the area of transportation. PPPs have been widely applied in transportation projects around the world to design, build, finance, operate, and transfer toll roads, highways, metros, airports, and railways [3]. PPPs have greatly contributed to the development of transportation networks for both the public and private sectors. The public sector can utilize capital, technologies, and management skills to pursue sustainable development by using PPPs [4]. Meanwhile, the private sector can obtain long-term returns and improve the reputation of companies in PPPs [5]. Moreover, transportation PPP projects can provide public facilities, services, and long-term partnerships for the general public with high levels of efficiency, quality, and convenience.

The goal of introducing the private sector into the field of infrastructure construction and operation is to improve the efficiency and effectiveness of public services and public good provision [6]. Therefore, public satisfaction, which has a close relationship with social welfare, is key to the success of a PPP project [7]. The general public and related public or private sectors surrounding projects are usually vulnerable and sensitive to change (e.g., landscape change, environmental impacts, and population migration) resulting from transportation projects, which can lead to many social problems [8]. Therefore, the public and private sectors face huge challenges in transportation PPP projects related to social changes. Many social problems, including poverty, health, education, traffic safety, gender equity, social safeguards, communicable diseases and trafficking, and labor employment, are influenced by transportation projects and the development of transportation systems [9]. Moreover, the involvement of the private sector in PPPs is likely to reduce the creditworthiness of transportation projects, which are usually built, operated, and maintained by the public sector, further decreasing the social value of transportation PPP projects [10].

Furthermore, social problems can be transformed into social risks if disputes related to social issues cannot be dealt with carefully and properly [11]. The concept of social risk refers to the risks that may influence the whole society and lead to social turbulence and social unrest in the forms of social tension and collective conflicts, which can spawn public confrontations, public protest and even violent conflicts [12,13]. Social conflict is the struggle of people in society using non-institutionalized or illegal modes of action to pursue their interests. These undisciplined actions definitely threaten social stability [14]. Social risks can be described as having the possibility of leading to social conflicts, which threaten social stability and social order, and causing further social crisis [14,15]. For a transportation PPP project, social risks can occur when stakeholders identify a project’s vulnerability from a social perspective. Social risks can destroy the reputation of transportation PPP projects, which may involve human rights, labor, or environmental sustainability [16]. Therefore, effective social risk management (SRM) needs to be carried out for transportation PPP projects. The SRM was defined by Holzmann and Jørgensen as “…public interventions to assist individuals, households, and communities better manage risk, and provide support to the critically poor” [17]. They also suggested three main risk management strategies (prevention, mitigation and coping) to deal with risk when the social risk events occur. Thus, the function of SRM for transportation PPP projects is to identify and subsequently mitigate, reduce and control the social risks that may occur before or during the process of these projects through corresponding risk governance strategies and plans [12].

Additionally, social risks can influence sustainability, as the benefits of multiple stakeholders can be negatively affected by social conflicts and crises. The WCED (World Commission on Environment and Development) initially defined sustainability as “Development that meets the needs of the present without compromising the ability of future generations to meet their needs,” which combines social, environmental and economic issues [18]. Thus, achieving the sustainable development goals (SDGs) of transportation systems requires a balance of the three pillars of sustainable development, which are the social, environment and economic sustainability [19]. According to the 17 SDGs set by United Nations [20], for the transportation systems, to achieve the social sustainability, the SDGs should include good health and well-being, quality education, equality, reduced inequalities, peace, justice, sustainable cities and communities among all the 17 goals; to achieve the environmental sustainability, the SDGs should include clean water and sanitation, affordable and clean energy, climate action, life below water, life and land; and to achieve the economic sustainability, the SDGs should include decent work and economic growth, industry, innovation and infrastructure, responsible consumption and production. Elkington indicated that the aim of social sustainability is to provide fair opportunities, encourage diversity, promote connectivity inside and outside the community, guarantee the quality of life and provide accountable governance structures and democratic processes [21].Therefore, identifying key social risk factors (SRFs) that could influence social sustainability and overall sustainability is crucial for a transportation PPP project and can be used as a product of modern civilization, globalization and equilibrium to improve the social value in a risky society [14]. The identification of SRFs for a transportation PPP project from the perspective of sustainable development can provide guidance on how to identify current and future requirements, determine the technologies and resources to meet those requirements, and balance the benefits of multiple stakeholders from the social dimension to achieve the SDGs.

Although great efforts have been focused on the environmental and economic aspects to achieve the SDGs of transportation projects, few studies have been conducted on the social aspects of transport. When the responsibilities of the public sector are shifted to the private sector in PPPs, the social risk should be given greater concern to improve the sustainability of transportation projects because the public interests and social protection are more vulnerable and sensitive.

Therefore, from the sustainable development perspective, how a transportation PPP project affects different stakeholders and society in its social dimension and how the relevant SRFs can be tracked and measured from multilateral levels should be explored to realize the effective SRM and measurement. Therefore, the SRFs identified and validated in this paper can be used to indicate how to implement SRM to cope with social changes and reduce negative social impacts in the lifecycle process of a transportation PPP project. In addition, the social resiliency of related urban transportation system can be further improved by effective SRM, and finally achieving the SDGs of transportation PPP project.

This paper begins with a literature review, followed by a discussion of the research methods adopted in this study and a conceptual model to identify possible 15 SRFs of transportation PPP projects under three SRF dimensions (economic, environmental and social dimensions) based on the theory of sustainable development. Furthermore, the relationships within the theoretical model are hypothesized. A questionnaire survey was conducted to investigate stakeholders’ opinions on these 3 dimensions and 15 SRFs. The results of survey show all the 15 SRFs under the SRF dimensions were significant for transportation PPP projects. Then, the hypothesized model of the relationships among SRFs, SRF dimensions, and social risks is tested and clarified by confirmatory factor analysis (CFA). The CFA results indicate that all SRF dimensions contributed to the change in the social sustainability of transportation PPP projects and that the classification of SRFs within the three SRF dimensions was precise. Finally, this paper provides some concluding remarks.

## 2. Literature Review

Transportation projects include roads, railways, airways, waterways, canals, pipelines, and terminals such as airports, railway stations, bus stations, warehouses, trucking terminals, refueling depots (including fueling docks and gas stations) and seaports. Transportation is significant because it promotes trade and communication among people, which is vital to the sustainable development of civilizations and urbanization [22]. Transportation projects have significant economic, environmental, and social impacts on stakeholders, societies and regions. In terms of the economic effects, investing in transportation projects will ultimately reduce the total transportation cost for the public, save time, and reduce traffic congestion, etc. For the environmental effects, there is a strong correlation among transportation projects, urban population density and per capita energy consumption. However, the construction and operation of transportation projects will also bring negative environmental effects due to multiple pollutions during the lifecycle process [8]. For the social effects, transportation can ensure that all members of society are able to travel and all stakeholders can be engaged and have the possibility of meeting other people in the stage of construction and operation, which could be related to poverty, health, education, traffic safety, gender equity, social safeguards, communicable diseases and trafficking, and labor employment [23].

**Figure 1:**
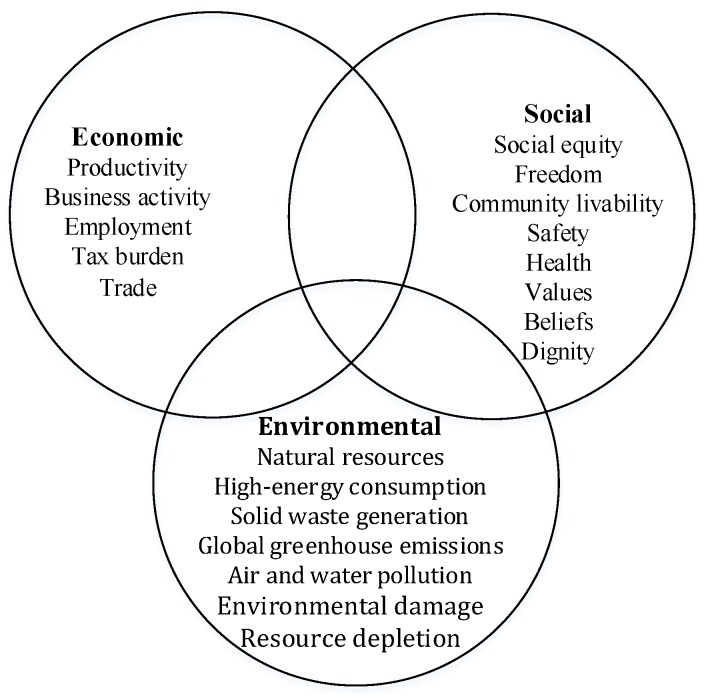
Three dimensions of SDGs for sustainable development of transportation.

For the sake of generating a balance of economic, environmental, and social impacts, sustainable transportation has been proposed in many prior studies [24,25]. Kennedy et al. indicate that the proper establishment of four pillars—the effective management of land use and transportation; equitable, efficient, stable funding; strategic infrastructure investments; and attention to neighborhood design—are required to achieve more sustainable transportation in the process [26]. Many prior studies have focused on the environmental and economic aspects of SDGs for transportation projects. Xue proposed that environmental sustainability be emphasized to prevent unnecessary consumption of natural resources (especially non-renewable ones) and mitigate high-energy consumption, solid waste generation, global greenhouse emissions, external air and water pollution, environmental damage, resource depletion, etc. [8]. Economic sustainability cares more about productivity, business activity, employment, tax burdens and trade. It strives not simply for material wealth (increased quantity) but also for social welfare outcomes (increased quality) [27]. Meanwhile, social sustainability is also an important part of the sustainable development of transportation [25]. However, the social dimension of sustainable development is widely accepted, which means, to be exact, that there is no clear definition or consent. Usually, narrowly defined social sustainability pays attention to social equity, freedom, community livability, safety, people’s health, values, beliefs, dignity, perceptions about society, etc. [27,28]. The concepts of social safeguarding, social capital, social protection, social value, social cohesion, social inclusion and social exclusion, which overlap, always connect with a broader concept of social sustainability [29]. As shown in Figure 1, each dimension of SDGs can be divided into a specific category. However, these three dimensions can overlap with each other [27]. For example, air pollution impacts the environment (environmental sustainability concerns), which also affects people’s health (social sustainability concerns) and living costs (economic sustainability concerns). Moreover, according to the aforementioned concepts, the goals of sustainable development (i.e., SDGs) of transportation PPP projects should integrate the economic, environmental, and social impacts at the same time [25,27].

Furthermore, PPPs are critically important for meeting the challenges proposed in the 2030 Agenda for Sustainable Development according to the working papers by United Nations, which also further demonstrates that PPPs can be an institutional tool with the sustainable goals of ensuring monetary value, minimizing potential financial risks and improving accountability [30]. Therefore, from the social dimension, social risks in a transportation PPP project can be viewed as possible threats to the SDGs. Transportation PPP projects can suffer from the complexity of the sources, as well as the forms and effects of these risks, which could make sustainable development difficult to be achieved [31]. Different from the traditional DBB (Design-Bid-Build) procurement method in transportation projects, which public services and goods are delivered by public sectors, private sectors in transportation PPPs are responsible for designing, building, financing, operating and maintenance, which greatly increase the difficulties and complexities [32]. Thus, many prior studies have made great efforts in the risk management of transportation PPP projects, including risk identification, evaluation and management [15]. Related studies mainly include political risks, interest risks, inflation risks, residual value risks, operation risks, and market risks. However, few studies have focused on the social risks on PPP projects due to their more serious and complex environment. To date, many research works related to social risk have been implemented in the field of construction management. Prior studies focused on three aspects relevant to social risks: the identification, assessment, and management of SRFs for construction projects [11]. In the context of transportation PPP, studies on SRFs should pay more attention to stakeholders, relationships, interfacing, culture, and the environment based on the perspective of all stakeholders, including owner, consultants, contractors, employees, the general public, and the community, to achieve sustainable development [33].

To mitigate and control the social risks of transportation PPP projects, local governments should establish an SRM system [11]. SRM is an important way for transportation PPP projects to reduce social vulnerability, enhance smooth consumption, improve social equity, smooth household welfare, and reduce poverty [17]. Three main strategies for SRM, prevention, mitigation and coping strategies, were suggested by Holzmann and Jørgensen [17]. In the project management of transportation PPP projects, social risks are closely related with stakeholders, not only the traditional participants (i.e., owners, contractors and employees) but also the public and community [33]. Therefore, SRM in such projects also reflects the features of social responsibility to address the environmental and social impacts of projects’ activities. In addition, social acceptance is one focus for SRM of the transportation PPP projects, and the level of the social acceptance depends on how the projects influence the social stakeholder groups’ benefits and impacts from a long-term perspective [34].

The SRF is a measure used in SRM to show how dangerous an activity is from a social perspective. The factors that influence the achievement of sustainable goals should be identified in detail. Many key SRFs should be identified to help both the public and private sectors know the key factors that can provide early warnings for potential future losses [35]. As mentioned by Holmquist, SRFs can provide raised risk awareness, early warning indicators, proactive risk management, and decreased losses that are expected or unexpected [36]. Usually, case studies and empirical studies are adopted to analyze the social risks in different situation. He et al. conducted a comprehensive case study on the decision-making process, public opinions, and protest actions regarding the planning and location selection for the Beijing–Shenyang high-speed railway from 2008 to 2013 [37]. Franks et al. used an empirical study to estimate the cost of conflict and identify conflict as an important means translating social risks into business costs [38]. Structural equation modeling was used by Wang et al. to study how the public responds when facing the social impacts of construction projects [39]. The factor analysis method is also considered as an effective approach to address social risks [40].

According to an extensive literature review, this study focuses on exploring various factors associated with the stakeholders and lifecycle process of transportation PPP projects leading to the undesirable deviation of sustainable development. SRFs not only address the process of estimating the social consequences that may be derived from transportation PPP project development but also recognize the stakeholders’ perceptions of social risks from the perspective of sustainable development. This study integrates a case study and an empirical study to identify the key SRFs and observe their interactions.

## 3. Research Method

**Figure 2:**
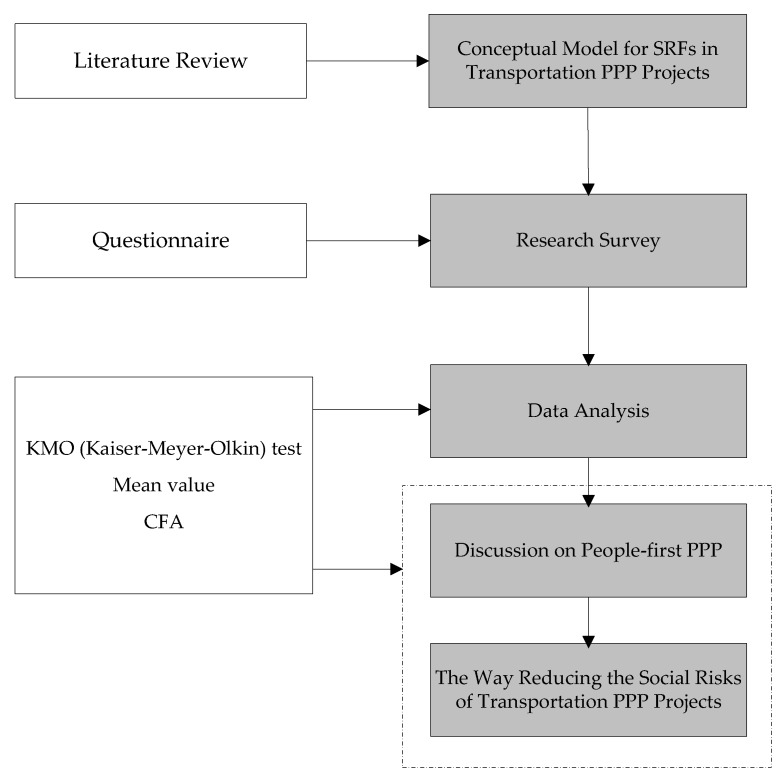
The research method adopted in this study.

A hybrid research method was employed in this study, as shown in Figure 2. First, based on a review of previous research results, a conceptual model is proposed using the theory of sustainable development. In addition, the potential SRFs that could lead to the social risks of transportation PPP projects are identified according to the proposed conceptual model. Then, a questionnaire was conducted to collect data to investigate the significance of the identified SRFs for transportation PPP projects. Based on the data collected from the survey, the SRFs were tested using multiple statistical analyses and the mean value to validate the survey results and evaluate the perceptions of SRFs. Confirmatory factor analysis (CFA) was further performed to test whether the theoretical model was consistent with the survey data and the relationship between SRFs was clearly defined. In addition, the contribution to the generation of different SRFs and SRF dimensions is discussed based on the significance level and factor loadings. CFA is aimed at not only detecting and explaining SRFs but also improving the present state of SRF management in actual transportation PPP projects and developing helpful social risk management in PPPs for future use. Furthermore, a people-first PPP method to reduce social risks is proposed to enhance the social sustainability of PPPs and achieve the SDGs of transport system.

A structured survey of the public sector, private sector, general public and researchers (related to transportation PPP projects) using a stratified sampling method was conducted from October to November 2016 to investigate the opinions of professionals and researchers on the SRF dimensions and SRFs of transportation PPP projects. All respondents of the survey are familiar with transportation projects, and they are the main stakeholders of transportation PPP projects. A total of 300 questionnaires were sent out to the respondents, and 196 valid responses were received. The effective return rate was 65.3%, which was acceptable and sufficient for our analysis. The information of the respondents is listed in Table 1.

The questionnaire was composed of two parts. The first part attained a brief overview of the respondents and their PPP projects. In the second part, the respondents were asked to use a five-point Likert scale to assess the relative significance of SRFs in Chinese transportation PPP projects. Through the second part, respondents’ opinions about the importance of each social risk factor were elicited. The ratio intervals of the Likert scale were as follows: (1) Can be ignored or not important; (2) Possibly important; (3) Important; (4) Very important; and (5) Most important. A five-point Likert scale can help interviewees clarify their views of agreement or disagreement on the importance of different factors [41].

In addition, the interviewees include officials from the public sector, managers from the private sector, the general public and related researchers from universities (see Table 1). The size of the sample was considered adequate for the purposes of data analysis when compared to other studies in the field of PPPs [42]. Most of the respondents worked for the government, the private sector, or research institutes with more than 5 years. The work experience of the respondents indicates that they have a good awareness of transportation PPP projects, which further ensured the reliability of the data. The survey data show that 34.69% of respondents were from academia, and 31.63% of respondents were from government. The researchers have rich theoretical knowledge of social risk, and the government is responsible for the management of social risks in transportation PPP projects to achieve the social sustainability of society. Therefore, the majority of the respondents were from the government or researchers, which can make the responses more reliable.

## 4. Sustainable Development-Based Conceptual Model and SRF Identification

### 4.1. Proposed Conceptual Model

As mentioned above, social sustainability is an important part of the SDGs of transportation [25], and the sustainable development of transportation PPP projects should integrate economic, environmental, and social impacts at the same time [25,27]. Thus, these impacts can affect social sustainability from different perspectives. In addition, for a transportation PPP project, the ultimate objective is to achieve VfM (Value for Money) for a public service and product. The definition of VfM is the maximum achievable outcome from the development of a transportation PPP project [43] In a new built transportation PPP project, the VfM objective should reflect the government’s overall strategic plan and mission objectives, the private sector’s long-term development and payoff strategy, and the general public’s requirements of quality public facilities and services [44]. Therefore, the achievement process of VfM for transportation PPP projects can also be affected by economic, environmental, and social impacts. Overall, the pursuit and realization of social sustainability and VfM should be closely related to economic, environmental, and social impacts. Because sustainable development is the final goal of transportation projects, these three impacts can affect the realization of SDGs through their effects on social sustainability and the VfM of the projects.

Traditionally, social sustainability can be considered as the degree of satisfaction based on the perspective of social justice, human dignity and participation from a sociological standpoint, according to Littig and Griessler [28]. This concept emphasizes the importance of “work” and “needs” and stresses the relationships between nature and society. Moreover, Weingaertner and Moberg suggest that social sustainability should improve the quality of life for all people, with the integration of diverse groups of different cultural and social background [45]. In this case, the critical elements for social sustainability (S_1_–S_13_) presented in Table 2 can be thoroughly selected from a review of prior literatures, with the purpose of finding key aspects and analyzing the main features of social sustainability, rather than being a never-ending list. The references of these critical elements have different focuses and scopes. Therefore, to keep the list short and focused, based on their definitions from the perspective of social sustainability, these critical elements can be divided into two levels, i.e., the project level and the system level. The project level emphasizes the influence of a project on the people’s interests and social stability, and these elements are closely associated with people living in the project area and the project related organizations’ activities [46]. The system level emphasizes the effects of the transportation system or urban system on the society and individual, which affects a wider range, and the elements of this level focus on the present and the future values and externalities (both positive and negative) of the system [19,25]. For example, “Governance (S_3_)” targets particular steps in the policy-making process such as options appraisal, decision making, and/or implementation [47]. Thus, “Governance (S_3_)” should be classified as the element at the system level. “Health and safety (S_4_)” is defined as a state of complete physical, mental and social well-being and not merely the absence of disease or infirmity [45]. This element should be categorized into both the project level and the system level. This study focuses on social sustainability at the project level. The goal of PPPs should also be considered when discussing the goal of sustainable development of PPP projects, which is the value for money (VfM). Therefore, S_1_ and S_4_–S_12_ in Table 2 are selected as critical elements of social sustainability for this study due to their focus on social sustainability at the project level and with the consideration of VfM.

**Figure 3:**
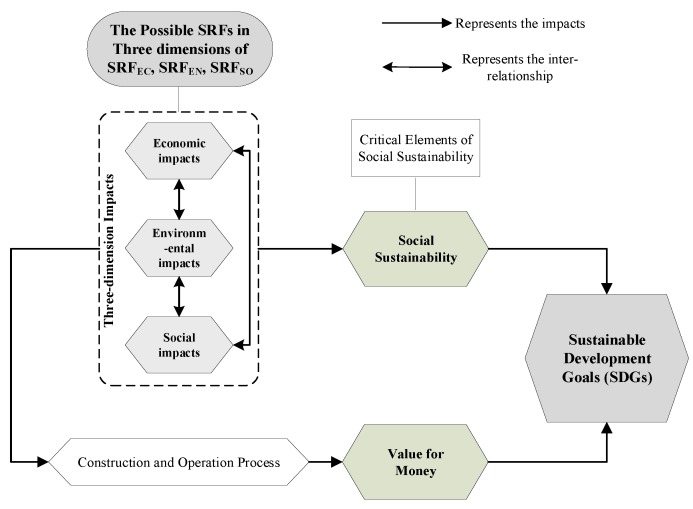
The conceptual model for SRFs in this study.

The conceptual model of this study is shown in Figure 3 and can be used to further identify the possible SRFs of transportation PPP projects. As mentioned above, to finally achieve the SDGs of transportation PPP projects, the realization of social sustainability and VfM is the premise. However, the three impacts (i.e., economic, environmental, and social impacts) will have negative effect on social sustainability and VfM during the project process. Thus, through the analysis of the three impacts on stakeholders and societies during the construction and operation process of transportation PPP projects and the critical elements of social sustainability selected above in Table 2, the possible SRFs of transportation PPP projects can be identified from the economic, environmental, and social dimensions. These SRFs of transportation PPP projects can be described as the factors that negatively influence the achievement of social sustainability and VfM of transportation PPP projects, which can further affect the realization of SDGs of a transportation PPP project.

### 4.2. Identification of Possible SRFs

Based on the conceptual model in Figure 3, by analyzing the three-dimension impacts on social sustainability and VfM during the construction and operation process of a transportation PPP project, and combing with critical elements of social sustainability at the project level (i.e., S_1_ and S_4_–S_12_ in Table 2), the possible SRFs of transportation PPP projects were identified from the economic dimensions (SRF_EC_), environmental dimensions (SRF_EN_) and social dimensions (SRF_SO_) as shown in Table 3.

#### 4.2.1. SRFs in the Economic Dimensions (SRF_EC_)

This dimension focuses on SRFs from the economic perspective to find the factors that can influence the critical elements of social sustainability for transportation PPP projects. In this dimension, the five factors (SRF_EC-1_–SRF_EC-5_) are related to tolls, compensation, construction and operation costs. A high toll (SRF_EC-1_) may negatively influence people’s equal access to transport services provided by transportation PPP projects [53]; therefore, the equal opportunities (S_1_) and human rights (S_7_) of social sustainability can be affected. Meanwhile, the compensation for land acquisition (SRF_EC-2_) will strongly influence human rights (S_7_) and the satisfaction of the general public [11]. Community involvement and development (S_12_) may also be negatively influenced in the process of land acquisition [14]. The indigenous rights of residents (S_8_) near the projects can also be influenced [54]. Furthermore, many problems during construction and operation, including construction delay (SRF_EC-3_) and maintenance and too much frequent repair (SRF_EC-4_), will increase the costs of construction and operation, which can increase the prices for the general public and the payments for government [55]. Construction delays and frequent maintenance can also affect the social dimension of sustainability for transportation PPP projects, which may lead to public opposition due to a period of decreased provision of public services (S_1_, S_7_) [4]. Hence, the satisfaction of the general public and government will be reduced. Moreover, the introduction of the private sector can influence employees in the context of PPPs, which may reduce employment opportunities and change the salary of employees (SRF_EC-5_) to improve the efficiency and profits [56]. Therefore, employment (S_5_), social security (S_6_), human rights (S_7_) and fairness labor rights (S_9_, S_10_) can be weakened.

#### 4.2.2. SRFs in the Environmental Dimensions (SRF_EN_)

This dimension focuses on the SRFs from the environmental perspective to find the factors that can influence the critical elements of social sustainability for transportation PPP projects. Transportation PPP projects are typically built both within urban and rural areas. Construction sites may produce multiple types of pollution, including air, water, soil, light and/or noise pollution (SRF_EN-1_, SRF_EN-2_, and SRF_EN-3_). Construction activities that cause air pollution (SRF_EN-2_) include land clearing, diesel engine operation, demolition, burning, and using toxic substances [57]. Meanwhile, surface water runoff and groundwater (water pollutions, SRF_EN-2_) near the construction site may be contaminated by various materials used in the construction project [57]. Construction sites produce a large amount of noise (noise pollutions, SRF_EN-1_), mainly from vehicles, heavy equipment and machinery [58]. In the operation and maintenance stage, air, water, soil, light and/or noise pollution can also occur due to traffic flows and facility users. Therefore, pollution, which can affect homeowners around the site, project owners, workers in the project, and users of projects, should be addressed in a transportation PPP project. In this case, the human rights (S_7_) of residents near the projects to enjoy a good environment can be affected. In addition, the health and safety (S_4_) of residents near the projects and workers in the projects may be reduced by environmental SRFs [59]. The local environment quality and employment rate (S_5_, S_9_) are also closely related issues. These environmental SRFs can have impacts on the satisfaction of different stakeholders.

#### 4.2.3. SRFs in the Social Dimensions (SRF_SO_)

This dimension focuses on SRFs from the social perspective to find the factors that can influence the critical elements of social sustainability for transportation PPP projects. Land acquisition (SRF_SO-1_) will not only have economic impacts but also influence the employment of residents and their rights (S_5_, S_6_, S_7_, S_9_, S_10_) [11]. During the construction process, safety (SRF_SO-2_) is always an important problem that can lead to the injury and death (heath and safety, S_4_), which can result in inequality (equal opportunities, S_1_) for the people in the accidents, strongly influencing the rights of survival and development (human rights, S_7_) [60]. In addition, social security (S_6_) can be influenced by safety [61]. It is important that the damage to culture heritage (SRF_SO-3_) or the human landscape during the construction and operation process would obviously reduce social cohesion and the social value of transportation PPP projects [62]. Thus, the social security (S_6_), human rights (S_7_) and indigenous rights (S_8_) of residents will be affected. Moreover, cultural heritage (S_11_) and community involvement and development (S_12_) can be negatively influenced. Public services provided by transportation PPP projects can be negatively affected by low user fees (SRF_SO-4_), which will influence the satisfaction of the public and private sectors. In this case, users’ equal opportunities (S_1_) and the fairness (S_10_) will be limited due to the poor public services. Traffic congestion (SRF_SO-5_) is a common problem for transportation projects and can affect the equal opportunities (S_1_) for users to obtain quality public services and even the physical and psychological health of users (S_4_, S_6_) [63]. Traffic congestion can also bring more carbon emissions and energy consumption [39], which may influence the development of the surrounding community (S_12_) and the protection of cultural heritage (S_11_) because of traffic pollution [64]. In the operation process of transportation PPP projects, repairs and maintenance are regular work. When untimely repairs and maintenance occur, the performance level of transportation project decreases, which influences stakeholders’ satisfaction in the area of equal opportunities (S_1_) to obtain public services and a high-quality community living environment [55]. Sometimes, quality problems may even cause accidents and bring harm to the health and safety (S_4_) of people [65]. Moreover, the quality failures (SRF_SO-6_) of public facilities and services, which may influence the satisfaction of different stakeholders, should receive more attention [66]. Social security should be addressed when serious quality problems occurs. Furthermore, problems related to social security (S_6_) can arise from unemployment due to land acquisition, construction safety and accidents, damage to cultural heritage, traffic congestion and salary changes. The degree of adequacy of facilities in the surrounding region (e.g., school, hospital, supermarket, etc.) should have significant influences on daily lives and wellbeing (S_6_). Inadequate facilities surrounding the projects (SRF_SO-7_) can reduce the equal opportunities of stakeholders to enjoy convenient services and have a negative impact on community development (S_12_).

### 4.3. Hypothesized Relationships in the Conceptual Model

The conceptual framework as shown in Figure 3 is a representation of the social risk in transportation PPP projects and is the basis for exploring SRFs. PPPs are aimed at providing the value of public products and services, including the satisfaction of the general public, efficiency, effectiveness, the sustainable development of local economy and society, and people’s life quality. Therefore, any changes in PPP projects that affect the achievement of social sustainability and VfM for public products and services will inevitably reduce the social values or social sustainability of transportation PPP projects, and ultimately affect the realization of SDGs of transport system. Based on these relationships, a hypothesis can be reached. The social risk of transportation PPP projects can be measured by the conceptual model, where all SRF dimensions can contribute to change s in social values or social sustainability in transportation PPP projects. As shown in Figure 3, all SRF dimensions contribute to the social values or social sustainability of transportation PPP projects from different perspectives. They all affect the social value of projects even though their contributions and approaches are different. Furthermore, the classification of these dimensions in the model reflects the features of social risk in PPPs. In addition, the survey data should provide an empirical evaluation of the factors arranged in different dimensions. The factors should be categorized in the above-mentioned dimensions according to their impacts on social values or social sustainability in transportation PPP projects. However, some factors have economic and social impacts on social values or social sustainability in transportation PPP projects at the same time. Therefore, the analysis of the survey data will help to identify which dimensions these factors influence more.

## 5. Descriptive Analysis

An analysis of reliability was conducted to test and verify the survey data’s internal consistency of variables. The Cronbach’s Alpha value was 0.825. The value was greater than the threshold recommended by Nunnally [67], who suggested that a reliability of 0.70 or higher should be sufficient in an early study on a construct’s prediction tests or hypothesized measures. The Cronbach’s value was derived for each indicator. Based on the Cronbach’s Alphas value, the opinions of the different participants on all SRFs were similar, laying a foundation for the development of further studies.

As shown in Table 4, the mean values ranged from 3.93 (Construction delay) to 3.37 (Quality failures) for the 15 factors. None of them were “not important” (less than 1.50) or “extremely important” (greater than 4.50), which indicated that all the 15 factors were significant. There were few differences in the views on the factors for different stakeholders, according to Table 4. Most of the SRFs’ standard deviations (SD) were less than 1, which shows that the scores of respondents were relatively consistent and values for SRFs in this study were acceptable for further research.

According to the survey results (Table 4), the mean values of 6 SRFs from the three SRF dimension are higher than 3.7. Among these indicators, the highest mean value was Construction Delay (3.93) from SRF_EC_ (Economic Dimension), which has a strong impact on the economic development and social change of transportation PPP projects. The second-highest mean value was Noise Pollution (3.86) from SRF_EN_ (Environmental Dimension), which means the environmental impacts were addressed by different stakeholders. The same mean value was obtained by Inadequate Compensation for Land Acquisition and Salary Change of Employees in Alternative Industries from SRF_EC_ (Economic Dimension), which was 3.85 ranking the 3rd in all SRFs. These two SRFs represent the benefits of stakeholders in the projects and out of projects. Another SRF, SRF_EC_ (Economic Dimension), i.e., High Prices (3.77) for end users in the transportation PPP projects, was obviously considered as very important by stakeholders. Another important SRF was Unemployment due to land acquisition (3.73), SRF_SO_ (Social Dimension). Land acquisition is not just an administrative means through which land is converted from collective-owned land to state-owned or SPV (special purpose vehicle)-owned land in China; it is also a process of huge life changes for the losers of lands.

## 6. Confirmatory Factor Analysis

### 6.1. CFA Methods

**Figure 4:**
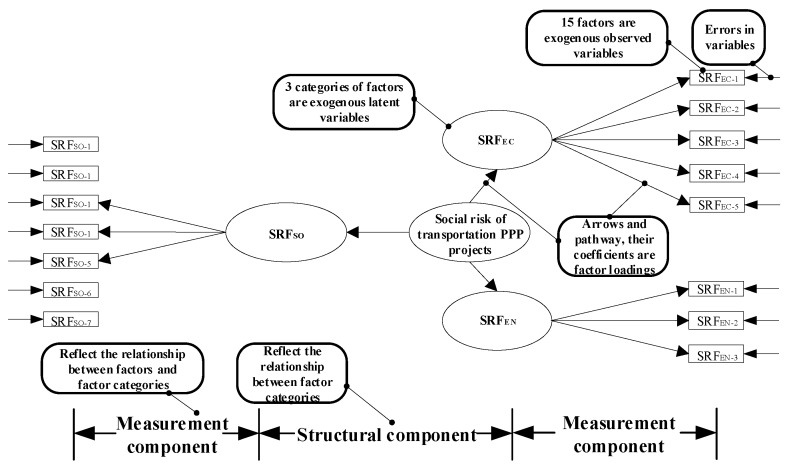
The hypothesized model for CFA.

As suggested by the hypothesis and shown in Figure 4, all SRF dimensions and factors contribute to the change of social values or social sustainability in transportation PPP projects (The conceptual model, Figure 3, was converted to a hypothesized model, as shown by Figure 4). To test if this SRF model fits the empirical data of the observation group as predicted, a CFA was conducted. Our research used a Likert-type scale as a response format. Likert scale data, as an ordinal scale, may violate the assumption of multivariate normality of the observed data in CFA. It is expected that the observed data follow the normal distributional assumption to obtain reasonable results. In fact, the distribution shape of variables in Table 4 indicates that the observed data basically accorded with a normal distribution, and the value of Cronbach’s alphas also verified the data’s reliability. In addition, the results of Kaiser-Meyer-Olkin (KMO) and Bartlett’s Test of Sphericity showed a high overall validity of the survey data. Thus, the survey data of this research was suitable for performing CFA.

Based on prior analytic research, CFA, which is a special factor analysis and a verification technique, can test if the survey data fit the hypothesized measurement model as presented in Figure 4. The proposed model includes endogenous observed variable (Social Risk), exogenous latent variables (SRF dimensions, i.e., SRF_EC_, SRF_EN_, and SRF_SO_), exogenous observed variables (SRFs, i.e., SRF_EC-1_, SRF_EC-2_, …, SRF_SO-7_), and errors in the variables and pathway coefficients (factor loadings). The latent variables that cannot be directly observed were measured with corresponding exogenous observation variables (SRFs). The straight line from the latent variables (SRF dimensions) to the corresponding observed variables indicates the cause-effect on the observed variables (indicators). The factor loadings on the straight lines represent the relationship of indicators with their associated latent variables.

Path diagrams are shown in Figure 4 to illustrate the relationships. Different variables’ relationships in a CFA can be complicated. In the path diagrams, ovals and rectangles separately represent the latent and measure variable, and arrows are used to link the variables and represented causality. The one-headed arrows signify the regression relationships and the direction of the arrow implies the direction of influence. The double-headed arrows indicate inter-correlation between the variables. The initial model shows the relationships between the SRFs and factor packages (SRF dimensions). To represent the hypothesized impact’s direction, one-headed arrows are used to connect the three factor packages and their corresponding factors.

The hypothesized model, as shown in Figure 4, was built to compute the population covariance matrix, which was compared to an observation covariance matrix when the CFA was conducted. The above SRF model was verified by using CFA to remove unimportant indicators. The SRFs that had low factor loadings (contribution to the social risk) were removed to achieve the optimal model, through which key SRFs could be identified. In addition, many SRFs (e.g., SRF_EC-3_ and SRF_EC-4_), as mentioned before, may have economic and social impacts on the change of social value or social sustainability for transportation PPP projects at the same time. CFA can help to test which factors should belong to which dimensions by finding an optimal model according to statistical analysis. 

A priori and certain number of item-loading patterns and indicators can be put forward by an optimal model. By comparing with an actual data set, a model’s sufficiency can be determined. According to Figure 4, the optimal model can be compared with the initial model systematically, which provides a direct test of the hypothesized superiority of the optimal model. A key issue related to every CFA is how to assess the overall model fit. To illustrate that the specified model is not a null mode, the chi-square (χ^2^) statistic is the indicator that is most widely used to assess a specified model. To avoid problems in relation to dependence on the sample size, diversities of indexes from different families were included in the evaluation of the model. These include the χ^2^/degree of freedom (Df), the comparative fit index (CFI), the normal fit index (NFI), and the root mean square error of approximation (RMSEA). According to Ng et al. [42], the suggested level of goodness-fit indices (GFIs) measures is shown in Table 5.

If some parameters are insignificant or some parameters should be moved to other dimensions, these tests, in this case, allowed the model to be improved and re-estimated. As a result, the improved model can be compared to the initial model, which used GFIs if some indicators were eliminated or moved to another dimension. Table 5 shows a summary of the recommended benchmarks for GFI and significance level tests adopted in this study.

### 6.2. Conducting the CFA to Analyze Data

First, the validity test was conducted to test whether a confirmatory analysis of factors can be conducted. The validity test mainly relies on Kaiser–Meyer–Olkin (KMO) inspection and the Bartlett Test of Sphericity of valid data, and the results are shown in Table 6. The KMO index of 0.841 (>0.5) and the significant Bartlett Test of Sphericity (Sig. 0.000 < 0.05) indicated that the overall validity of the questionnaire is high, and the confirmatory analysis of factors can proceed [68]. 

**Figure 5:**
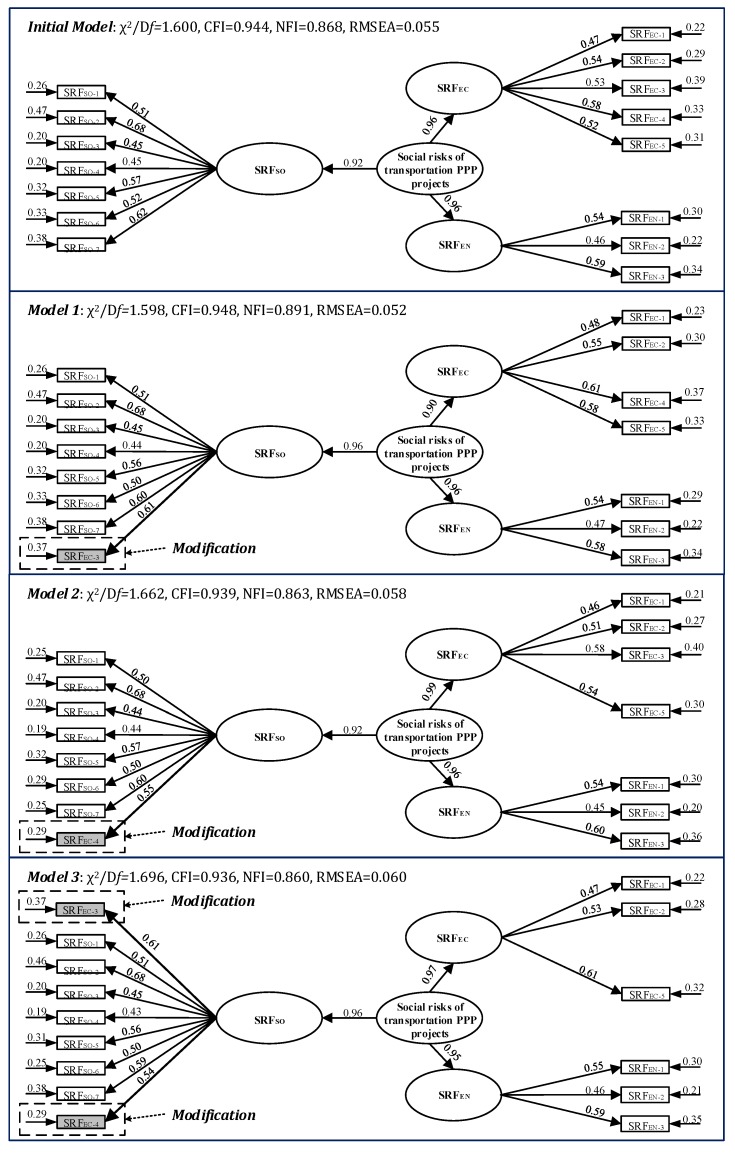
The estimations of proposed models by CFA.

Following the CFA, the variables and the errors among the variables are presented in Figure 5. Four models are presented, including the initial model, model 1 (moving SRF_EC-3_ to the SRF_SO_ dimension), model 2 (moving SRF_EC-4_ to the SRF_SO_ dimension), and model 3 (moving SRF_EC-3_ and SRF_EC-4_ to the SRF_SO_ dimension). As presented before, SRF_EC-3_ and SRF_EC-4_ can affect both the economic and social dimensions of the social sustainability of transportation PPP projects in China. CFA can help to test which relationship is better. The coefficients of the arrows and pathway (factor loadings) as shown in Figure 5 can present the causal effect statistically and in terms of the relationship of the SRF dimensions and SRFs influencing the social value and social sustainability of transportation PPP projects in China by the proposed model. The measurement and structural components are also shown in Figure 5, illustrating that the model reflects the relationships of the factors and factor packages directly.

CFA was used to test the initial model, Model 1, Model 2 and Model 3, and a parameter estimation and GFIs of the model as shown in Table 7 were produced to find the best model representing the appropriate relationships among social risk, the SRF dimensions and SRFs. The estimates of the pathway coefficients are shown in Figure 5. For the four raised models, the CFI value was good enough, and the value of RMSEA indicated that the requirements of further analysis can be met, according to Ng et al. [42]. According to Table 5 and Figure 5, the hypothetical Model 1 had relatively higher GFIs measure compared to the other models. Based on the estimation, Model 1 had the best model fit, as shown in Table 7. Therefore, Model 1 can be viewed as the most appropriate model according to the statistical analysis.

Based on Model 1 in Figure 5, all three SRF dimensions were significant and contributed to the generation of social risks in transportation PPP projects in China. All factors were found to be significant and correlated with their corresponding dimensions. The factor loadings from the three SRF dimensions to the social risks and from the factors to their corresponding dimensions are shown in Figure 5. Most of the factor loadings were greater than 0.50 or close to 0.50, which was considered adequate for estimation [69]. According to the CFA results for the four models in Figure 5, the proposed best model (Model 1) correlated relatively well with the observed data (RMSEA = 0.052). The three dimensions, factors, and their assumed relationships were confirmed by the correlation with empirical data. The social risks of transportation PPP projects can be described in terms of three perspectives, which includes the economic, environmental, and social effects on the change in the social value and social sustainability. A very good fit was shown between the improved model and the observed data when testing the best model (Model 1). The best model (Model 1) can be used to describe the relationships between social risks, the three dimensions, and the SRFs, providing an accurate model of SRFs.

### 6.3. Measurement Component of SEM Framework

The latent variables describing the economic impacts on social sustainability were measured by SRF_EC-1_, SRF_EC-2_, SRF_EC-4_, and SRF_EC-5_. All factors in this factor dimension contributed to the economic influences, but to different levels. The most important effect was SRF_EC-4_ (Frequent repairs in the operation). Though this factor did not achieve the highest score in the questionnaire survey (mean value = 3.61), the results of the CFA indicated that the frequency of maintenances will increase the operation costs and strongly influence the life of the related stakeholders in transportation PPP projects (SRF_EC-4_→SRF_EC_, 0.61). The next most important factors were SRF_EC-5_ (Salary change of employees in alternative industries, 0.58) and SRF_EC-2_ (Inadequate Compensation for Land Acquisition, 0.55). These two factors received the third highest score in the questionnaire survey. The results of CFA and the mean value indicate that the construction and operation of transportation PPP projects can provide new opportunities for residents surrounding the projects. In addition, the improvement of traffic conditions also improve the employment position, which can increase the competition of employees [56]. At the time, the economic problems in land acquisition were highly addressed by stakeholders. In reality, the introduction of private sectors in transportation PPP projects in China will influence the attitudes of the general public on social justice, which can lead to many social conflicts. The least important factor was SRF_EC-1_ (High Prices, 0.48), with the fifth high mean value in the questionnaire survey (mean value = 3.77), which meant stakeholders may be more concerned about the impacts of high prices on the economic dimension of sustainability. However, the case from the Hong Kong Western Harbour Crossing also indicated that a too frequent price adjustment would arouse the dissatisfaction of stakeholders [70].

The latent variables describing the environmental impacts on social sustainability were measured by SRF_EN-1_-SRF_EN-3_. Factors in this factor dimension had significant contributions to the environmental influences to varying degrees. According to the CFA results, SEF_EN-3_ (Water Pollutions, 0.58) received the highest factor loadings in this factor dimension, which means the discharges from construction sites or operation process should be properly handled to reduce the pollution threat to human beings. Another important factor was SRF_EN-1_ (Noise Pollutions, 0.54), which received the second highest mean value in the questionnaire. Preventive measures should be adopted to reduce the amount of noise in the neighboring community. Noise may influence the health of different stakeholders adversely, including effects such as stress, high blood pressure, sleep disturbances and even hearing loss [59]. Moreover, Air Pollutions (SRF_EN-2_, 0.47) should not be ignored. In the construction process, more effective methods to reduce air pollutions should be used, because building sites generate high levels of dust (typically from wood, concrete, stone, cement, silica) and this can carry over large distances for a long time. Traffic jams, which increase vehicle emissions and degrade ambient air quality in the operation process, should be well controlled [71]. 

The latent variables describing the social impacts on social sustainability were measured by SRF_SO-1_-SRF_SO-7_, and SRF_EC-3_ (Rename it as SRF_SO-8_, Construction Delay). Among these eight factors, SRF_SO-2_ (Construction Safety and Accidents, 0.68) received the highest factor loading within this factor package, which indicated that safety risks in PPP projects could lead to strong challenges and criticisms by society. Another problem in the construction period, SRF_EN-3_ (SRF_SO-8_) (Construction Delay, 0.61), received the second highest factor loading from the perspective of social impacts. This factor initially was in the dimension of economic impacts, which means that it could strongly affect social sustainability from the perspective of economic impacts. However, the survey data integrating the opinions of different stakeholders indicated that construction delays might have more important social impacts on the social sustainability of transportation PPP projects. Social anxieties and complaints could arise due to any delays in the provision of public goods and services in transportation PPP projects [4,55]. The significance of human rights protection and public service quality were highly addressed in this dimension. The rights to work (SRF_SO-1_, Unemployment due to land acquisition), to pass on humanistic spirit (SRF_SO-3_, Damages of Cultural Heritage) and to enjoy equitable public facilities (SRF_SO-7_, Inadequate Facilities Surrounding the Projects) should receive more attentions, which would increase the social value of transportation PPP projects. In these human rights-related factors, the demand for more public facilities was the most important factor. In addition, the quality of public services directly influences the attitudes of the general public through management on price adjustments (SRF_SO-4_, Poor Public Service due to Low Prices), traffic flow (SRF_SO-5_, Traffic Congestion), and construction and maintenance (SRF_SO-6_, Quality failures). Traffic congestion (SRF_SO-5_, 0.56) was the most significant factor in these public service quality-related factors, which means the emphasis of private sectors should turn from hard facilities to soft services to improve public satisfaction.

### 6.4. Structural Component of CFA Framework

The structural component of Model 1 is shown in Figure 5. All of these three dimensions were found to be important in Model 1. Therefore, the proposed classification into three dimensions is verified. According to the structural component of the CFA framework, the different SRF dimensions were all significant and all contributed differently to the change in social sustainability in transportation PPP projects. The difference can be reflected based on the factor loadings of different dimensions. The dimension of environmental impacts and social impacts both obtained the relatively higher loadings (0.96), which indicates that reducing social and environmental impacts is very important for the sustainable development of transportation PPP projects and can improve the social sustainability of transportation PPP projects. The responsibilities of private sectors in transportation PPP projects can greatly affect people’s lives in a direct way by providing accessibility to employment, healthcare, food and recreation facilities, and in an indirect way by making changes in the living environment, transportation conditions, and urban areas. Therefore, both of public and private sectors should know and understand the human rights impacts of their activities. The social risk assessment for transportation PPP projects need to consider both an environmental impact assessment (EIA) and social impact assessment (SIA), in which impacts of human rights are considered carefully. The economic impact dimension has a relatively low score compared to the other two dimensions (0.90). Actually, the economic impacts of PPPs have long been addressed by prior studies, including VfM, cash flow, and cost benefits. Compared to social and environmental impacts, the economic impact was less important when focusing on the changes in social sustainability, which means the social risks in transportation PPP projects were more related to social and environmental influences.

## 7. Discussion on Social Risk

This research showed that SRFs could affect social sustainability of transportation projects delivered by PPPs in China, as well as their contributions to the generation of social risks. The statistical analysis verified the significance of the 15 factors. An improved model that entailed adjusting the factor was finally determined. The interrelationships between different SRFs and different SRF dimensions were illustrated according to the CFA of the 15 factors included in the above model, for the sake of providing an effective foundation for the rational assessment and management of social risks in transportation PPP projects in China to improve social sustainability and social value. An important research finding is that people-first PPPs would be very helpful for reducing social risks.

To realize SDGs of transportation PPP projects, the key challenge is balancing economic and social progress with environmental considerations, according to our studies. According to Table 4, the factors with a higher mean value were in the economic dimension, which meant economic impacts were more important compared to social and environmental impacts. In contrast, the contributions of SRFs to the generation of social risks from a social perspective were larger than those from economic and environmental perspectives. Although there were differences for different data analysis results, different stakeholders shared a common sense on the essence of social risks of transportation PPP projects and related SRFs. 

In addition, the CFA analysis indicated that the generation of social risks could rely more on social impacts. According to Figure 5, The top 6 SRFs that contributed to the generation of social risks in transportation PPP projects were SRF_SO-2_ (Construction Safety and Accidents), SRF_SO-8_ (Construction Delay), SRF_SO-7_ (Quality failures), SRF_EN-3_ (Water Pollutions), SRF_EC-3_ (Frequent repairs in the operation), and SRF_SO-5_ (Traffic Congestion), among which four factors were from SRF_SO_ (social dimension). 

Therefore, the SRFs of transportation PPP projects can be used to help achieve the SDGs from social perspective. SRFs should be associated not only with economic improvement but also with major social transformation. Traditionally, the introduction of private sectors was done to improve the efficiency or value-for-money of public procurement and management [55]. However, the research findings indicate that urgent consideration and more action should be pursued through the adoption of PPP to realize a sustainable investment to “fit for purpose” for the long-term SDGs. In this case, people-first PPP is a clear statement that out of all the stakeholders, “people” should be the priority and main beneficiary. In the context of transportation PPP projects, the social responsibility of PPP should focus on facilitating effective construction and operation management to reduce the negative influences on the satisfaction of stakeholders and quality of life of surrounding communities. Another important issue for transportation PPP projects is to improve the values for the external stakeholders, including creating local and sustainable jobs, fighting environmental pollutions, promoting wellbeing and culture, and increasing access to public services.

People-first PPP represents the new generation of public management and services, which would help to further reduce social risks in transportation PPP projects. In addition to being effective tools to put assets “off the country’s balance sheet” and provide better services through VfM-based management, people-first PPP should not only focus on profits but also pay greater attention to quality investments that feature accessibility, equity, efficiency, effectiveness, and sustainability. Hence, many measures to reduce social risks of transportation PPP projects can be proposed.

- From the perspective of internal stakeholder management, the efficiency and effectiveness should be improved through lifecycle project management. Time and quality are the main components of The Iron Triangle, which are important not only for normal projects but also for PPP projects and have become inevitably linked with measuring the success of project management [55]. Avoiding construction delays (SRF_SO-8_) should be the most important mission in transportation PPP projects to reduce social risks, and this would help to provide public goods and services with high efficiency. Quality (SRF_SO-7_) is an emergent property of people’s different attitudes, satisfaction and beliefs, and it tends to change in the life cycle of the transportation PPP project [66]. Meanwhile, safety management (SRF_SO-2_) is viewed as an important index to measure the success of a PPP project. Additionally, important issues in transportation PPP project management are performing better operation and maintenance management. Periodic repairs and the improvement of maintenance quality (SRF_EC-3_) would be helpful to increase the satisfaction of stakeholders. In addition, management on traffic flow and price adjustment are unique features of a transportation PPP project and can face great challenges. In this case, detailed and careful studies on traffic prediction and pricing mechanism should be implemented to consider the requirements of different stakeholders. Dynamic monitoring or control for traffic flow, costs, revenue, and inflation, etc. are also essential during the construction and operation period and can provide effective information and responding mechanism for the dynamic external environments (SRF_SO-5_). Therefore, increasing efficiency and being effective are the primary measure from a project perceptive to reduce social risks in transportation PPP projects and should improve the productivity of existing assets, create savings, and make the public service work and deliver.
- From the perspective of external stakeholder management, access to public service and relationship management should be assured. Users and related residents around the project can be viewed as external stakeholders. First, increasing the access of essential services to people can reduce the possibility of social changes, decrease prices, improve the service quality, and enhance the configuration of public facilities. Furthermore, the human rights of external stakeholders should be highly prioritized when reducing the social risks of transportation PPP projects, including the rights to hold properties, enjoy clear water and air, experience reduced noise, obtain work, and take in culture (SRF_EN-3_). In reality, social risks are always accompanied by social changes. Hence, reducing the possibility of social changes can help to reduce the social risks. Social change does not occur at a constant pace. The forces bringing social change in a transportation PPP projects can be linked with all aspects of the social fabric due to the introduction of private sectors. The interactions among public sectors, private sectors, and the general public have changed the social structure of traditional transportation projects and brought about changes in social development. Therefore, raising compensation, creating more employment opportunities, protecting cultural heritage, changing demographic patterns, facilitating technological advances, etc. are possible ways of influencing social change, which may prevent a society influenced by transportation PPP projects from flexibility, fragmentation, polarization and differentiation in time and space. In addition, the development of transportation PPP projects could bring combinations of new networks, externalities and the breaking down of barriers, which can result in the concentration and unity of geography and society. However, the interests and benefits of different stakeholders may be diverse and widespread. In this case, the interactions between the changing social structure, travel behavior, land use, and environmental impacts (e.g., CO_2_ emissions) should be carefully examined.

## 8. Conclusions

This paper not only identified SRFs for transportation PPP projects but also tested the relationships between the SRFs, SRF dimensions, and social risks of PPP transportation projects. A conceptual model of SRFs was proposed and further developed through the analysis of a hypothetical relationship measuring transportation PPP projects’ social risks. A questionnaire survey was used to investigate stakeholders’ views on three SRF dimensions (i.e., economic, environmental, and social dimensions) and 15 SRFs that influence the social risks of a transportation PPP project. The results of the survey showed that all of the 15 SRFs were important (>3.00) and can be used to evaluate the social risks from the three SRF dimensions. The CFA method was also adopted to test whether the hypothesized model is associated with survey data. The results of the CFA on the improved Model 1 showed a good model fit, which indicated all SRF dimensions contributed to the change in social sustainability in transportation PPP projects and that the classification of SRFs within the SRF dimensions was precise (Hypothesis in this paper). Based on the mean value of SRFs obtained from the results of the survey, the most important factors for social risks that should be the balanced results among economic, environmental and social impacts include *construction delay*, *noise pollutions*, *inadequate compensation for land acquisition*, and *salary change of employees in alternative industries*, *high prices*, and *unemployment due to land acquisition*. The CFA results indicated that social and environmental impacts on the social sustainability of transportation PPP projects should contribute more to the generation of social risks. The social responsibilities of private sectors should emphasize safety management, time control, quality management, wastewater treatment, maintenance management, and traffic flow management.

In transportation PPP projects, the 15 SRFs provide a useful tool for distinguishing advantages and disadvantages from effective SRM and measurement. In addition, the CFA results provide a basis for long-term and sustainable development to reduce social risks and effectively meet the social value and social sustainability requirements of transportation PPP projects, which can facilitate the realization of SDGs. Therefore, people-first PPP was proposed to further reduce social risks in transportation PPP projects. From the perspective of internal stakeholder management, the efficiency and effectiveness should be improved through lifecycle project management. Increasing efficiency and being effective are the primary measure from a project perceptive to reduce social risks. From the perspective of external stakeholder management, access to public service and relationship management should be assured. Moreover, the interactions among public sectors, private sectors, and the general public should be highly addressed to reduce the possibility of social change.

A potential use of identified SRFs is to identify the weakness of SRM and to measure the social risks in PPPs, where different measurement methods can be adopted to monitor and calculate any specific changes of social value and social sustainability when conducting PPP projects. Moreover, identified SRFs can be used to implement effective SRM for transportation PPP projects to reduce negative social impacts and to improve the resiliency of the urban system.

Although this research on SRFs promotes further understanding of SRM in PPP projects, there are some limitations of the research. The causal relationships between the different SRF dimensions should be verified in future studies. The clarification of the relationships between different SRFs will promote how to effectively measure and reduce the understanding of SRFs in PPP projects, which means that further research should explore how best to apply the SRFs. Moreover, the relationships of economic, environmental, and social impacts on the change in the traffic flow, travel behavior, and total utilities of transportation PPP projects should be further clarified.

## Figures and Tables

**Table 1:** Profile of the respondents.

	Respondents		Valid Questionnaire	Percentage	
**The role of respondents**	Government officer		62	31.63%	
	Managers for private sectors		30	15.31%	
	The General public		24	12.24%	
	Financial institution		12	6.12%	
	Researchers		68	34.69%	
	Total		196	100%	
**The experiences of respondents**	**Experiences**	**In Industry**	**Percentage**	**In PPPs**	**Percentage**
	≤5 years	58	29.59%	91	46.42%
	6–10 years	80	40.82%	73	37.24%
	11–15 years	46	23.47%	27	13.78%
	≥16 years	12	6.12%	5	2.56%
	Total	196	100%	196	100%

**Table 2:** Critical elements of social sustainability.

No.	Critical Elements of Social Sustainability	Sources	Project Level	System Level	Is This Value for Money?
S_1_	Equal opportunities	[28,45,48]	√	√	√
S_2_	Education and training	[45,49,50]		√	
S_3_	Governance	[47,51]		√	
S_4_	Health and safety	[29,50]	√	√	√
S_5_	Employment	[25,50,52]	√	√	
S_6_	Security	[23,29,45]	√	√	√
S_7_	Human Rights	[27,48]	√	√	
S_8_	Indigenous rights	[50,52]	√	√	
S_9_	Labor practices	[28,45]	√	√	
S_10_	Fair operating practices	[23,45]	√	√	
S_11_	Cultural heritage	[29,50]	√	√	
S_12_	Community involvement and development	[27,29,45]	√	√	√
S_13_	Technology development	[45,48]		√	√

“√” in the table means the critical elements of social sustainability (S_1_–S_13_) belong to the corresponding level (i.e., project level/system level/is this value for money?).

**Table 3:** The identification of SRFs to achieve the social sustainability of transportation PPP projects.

Social Risk Dimensions	SRFs of Transportation PPP Projects		Critical Elements of Social Sustainability for Transportation PPP Projects									
			S_1_	S_4_	S_5_	S_6_	S_7_	S_8_	S_9_	S_10_	S_11_	S_12_
SRF_EC_ (Economic Dimension)	SRF_EC-1_	High Prices	√				√					
	SRF_EC-2_	Inadequate Compensation for Land Acquisition					√	√				√
	SRF_EC-3_	Construction Delay	√				√					
	SRF_EC-4_	Frequent repairs in the operation	√				√					
	SRF_EC-5_	Salary Change of Employees in Alternative Industries			√	√	√		√	√		
SRF_EN_ (Environmental Dimension)	SRF_EN-1_	Noise Pollutions		√	√		√		√			
	SRF_EN-2_	Air Pollutions		√	√		√		√			
	SRF_EN-3_	Water Pollutions		√	√		√		√			
SRF_SO_ (Social Dimension)	SRF_SO-1_	Unemployment due to land acquisition			√	√	√		√	√		
	SRF_SO-2_	Construction Safety and Accidents	√	√		√	√					
	SRF_SO-3_	Damages of Cultural Heritage				√	√	√			√	√
	SRF_SO-4_	Poor Public Service due to Low Prices	√							√		
	SRF_SO-5_	Traffic Congestion	√	√		√					√	√
	SRF_SO-6_	Quality failures	√	√								
	SRF_SO-7_	Inadequate Facilities Surrounding the Projects	√									√

“√” in the table means the SRFs can influence the critical elements of social sustainability.

**Table 4:** The mean values and rankings for transportation PPP projects in China.

Social Risk Dimensions	SRFs of Transportation PPP Projects		Mean Value	Ranking within the Dimension	Ranking	S.D.	Distribution Shape	
							Skewness	Kurtosis
SRF_EC_ (Economic Dimension)	SRF_EC-1_	High Prices	3.77	5	5	0.978	−0.819	0.632
	SRF_EC-2_	Inadequate Compensation for Land Acquisition	3.85	2	3	0.952	−0.528	−0.434
	SRF_EC-3_	Construction Delay	3.93	1	1	1.058	−0.918	0.257
	SRF_EC-4_	Frequent repairs in the operation	3.61	4	8	0.930	−0.491	0.020
	SRF_EC-5_	Salary Change of Employees in Alternative Industries	3.85	2	3	0.984	−0.645	−0.122
SRF_EN_ (Environmental Dimension)	SRF_EN-1_	Noise Pollutions	3.86	1	2	1.021	−0.684	−0.135
	SRF_EN-2_	Air Pollutions	3.46	3	14	0.941	−0.403	−0.147
	SRF_EN-3_	Water Pollutions	3.61	2	8	1.138	−0.426	−0.598
SRF_SO_ (Social Dimension)	SRF_SO-1_	Unemployment due to land acquisition	3.73	1	6	0.956	−0.620	−0.052
	SRF_SO-2_	Construction Safety and Accidents	3.58	4	11	0.955	−0.596	0.168
	SRF_SO-3_	Damages of Cultural Heritage	3.59	3	10	0.975	−0.278	−0.342
	SRF_SO-4_	Poor Public Service due to Low Prices	3.49	5	12	1.055	−0.331	−0.490
	SRF_SO-5_	Traffic Congestion	3.48	6	13	1.010	−0.442	−0.264
	SRF_SO-6_	Quality failures	3.37	7	15	0.899	−0.109	−0.103
	SRF_SO-7_	Inadequate Facilities Surrounding the Projects	3.63	2	7	0.933	−0.492	0.029

**Table 5:** The recommended level of goodness-fit indices (GFIs) measures.

GFIs (Goodness-Fit Indices)	Recommended Level of GFIs
χ^2^/degree of freedom (Df)	From 1 to 2
Comparative fit index (CFI)	0 (no fit) to 1 (perfect fit)
Normal fit index (NFI)	0 (no fit) to 1 (perfect fit)
Root mean square error of approximation (RMSEA)	<0.05 indicate very good fit (Threshold level = 0.1)

**Table 6:** Results of Kaiser-Meyer-Olkin (KMO) analysis and Bartlett’s Test of Sphericity.

Indicators	Values	
KMO measure of Sampling Adequacy	0.841	
Bartlett’s Test of Sphericity	Approx. Chi-Square	940.232
	Df (Degree of freedom)	105
	Sig. (significance)	0.000

**Table 7:** The measured values of goodness-fit indices (GFIs) for proposed four models.

GFIs (Goodness-Fit Indices)	Measured Values			
	Estimation for Initial Model	Estimation for Model 1	Estimation for Model 2	Estimation for Model 3
χ^2^/degree of freedom (D*f*)	1.600	1.598	1.662	1.696
Comparative fit index (CFI)	0.944	0.948	0.939	0.936
Normal fit index (NFI)	0.868	0.891	0.863	0.860
Root mean square error of approximation (RMSEA)	0.055	0.052	0.058	0.060
